# Identification of characteristics, risk factors, and predictors of recurrent LVAD thrombosis: conditions in HeartWare devices

**DOI:** 10.1007/s10047-020-01228-2

**Published:** 2020-12-18

**Authors:** Takayuki Gyoten, Michiel Morshuis, Sebastian V. Rojas, Marcus-André Deutsch, René Schramm, Jan F. Gummert, Henrik Fox

**Affiliations:** Clinic for Thoracic and Cardiovascular Surgery, Herz- und Diabeteszentrum Nordrhein-Westfalen, Universitätsklinik der Ruhr-Universität Bochum, Georgstrasse 11, 32503 Bad Oeynhausen, Germany

**Keywords:** LVAD, Thrombolysis, Pump thrombosis, t-PA, Surgical pump exchange, HeartWare

## Abstract

**Background:**

Redictors of repetitive left-ventricular assist device (LVAD)-thrombosis have not been studied yet.

**Methods:**

We identified predictors of recurrent LVAD thrombosis in HeartWare (HVAD) patients in a long-term study from 2010 until 2020. We included all patients with two or more thrombolysis treatments for repetitive HVAD thrombosis and effectiveness of thrombolytic therapy was defined as freedom from stroke, death, another HVAD thrombosis, or surgical device exchange within 30 days after the event. Study endpoints also include all-cause mortality and heart transplantation.

**Results:**

A total of 534 HVAD implantations have been screened, and 73 patients (13.7%) developed first HVAD thrombosis after a median of 10 months (IQR; 6–21 months). 46 of these patients had effective thrombolysis in 71.7% (*n *= 33/46). After a median of 14 months (IQR 4–32 months) follow-up, 17 patients (51.5%) had developed a second HVAD thrombosis and all were treated with t-PA therapy again, resulting in effectiveness in 76.5% (*n *= 13/17). The four patients with ineffective t-PA therapy underwent subsequent surgical HVAD exchange. Multiple Cox regression model analysis revealed time interval between HVAD implantation and first thrombosis as an independent risk factor of recurrent thrombosis (HR, 0.93, 95% CI 0.87–0.99, *p *= 0.031). Kaplan–Meier analysis at 3 year follow-up showed no significant difference in overall survival for recurrent vs non-recurrent thrombosis groups (log-rank test, *p *= 0.959).

**Conclusion:**

Recurrent HVAD thrombosis mostly appears within 12 months after first thrombosis. Systemic t-PA therapy for recurrent pump thrombosis seems safe, achieving comparable effectiveness rates to initial t-PA therapy. Survival does not differ between patients with or without recurrent HVAD thrombosis.

## Introduction

Long-term mechanical circulatory support with left-ventricular assist devices (LVAD) has increasingly become an established treatment option in advanced heart failure with improving outcomes during the past 2 decades [1, 2]. In clinical routine, indication for LVAD implantation is provided by progressive heart failure (HF) often but not exclusively with severely impaired left-ventricular function in the context of exhausted medical therapy [1, 2]. However, LVAD thrombus formation either in the inflow cannula, in the device body, or the outflow graft is one of the major complications that can lead to life-threatening low-flow or pump stop, implicating hemodynamic instability, hemolysis, renal or hepatic failure, or cerebral or peripheral thromboembolism [1–3].

The pathophysiology of LVAD thrombosis is a multi-factor proceeding deriving from (1) embolus formation secondary in the endocardial surface of the left ventricle when it comes to tardy blood flow, as well as clots formed in the left atrial appendage, or in ventricular debris originating from previous surgery; (2) insufficient anticoagulation or antiplatelet therapy; 3) inflow cannula malposition [3]. Incidence of LVAD thrombosis has been reported to emerge in 2–13% of adult LVAD patients with modern continuous-flow devices [3–7]. As a result, immediate action is necessary, and current scientific discussion is indecisive about the auspicious and best treatment strategy. Some proclaim surgical pump exchange to be the superior method of treatment, as all thrombus material can be removed, and a fully functional pump is reinstalled [8]. Surgery is associated with invasiveness and a redo procedure, connoting prolonged recovery periods and the risk of remaining detriment such as infection, organ injury, or failure. Others state systemic thrombolysis as the treatment of choice in LVAD thrombosis, but systemic thrombolysis is associated with bleeding incidence and uncoupling thrombotic events. No randomized-controlled trial has compared the two strategies yet, and in particular, nothing is known about promising handling of a recurrent LVAD thrombosis so far.

In case of hemodynamic instability, surgical LVAD exchange is the preferred method to overrule hypoperfusion and to prevent life-threatening LVAD stop. In a multicenter analysis of 21 patients with LVAD thrombosis, medical therapy alone in HeartWare HVAD was associated with success rates of only 48% and high amounts of complications such as hemorrhagic stroke (21%) and death (10%) [9]. This trial favored surgical LVAD exchange as the treatment of choice, even in patients with stable hemodynamics [9]. However, according to the Interagency Registry for Mechanical Assisted Circulatory Support (INTERMACS) report, surgical LVAD exchange is associated with high mortality too, and comparative poor prognosis with a 1-year survival after surgical LVAD exchange below 65% [10]. In addition to that, 1-year survival rate is even worse in patients with a second LVAD exchange procedure in recurrent LVAD thrombosis (10). In this context, many centers focus on improving their management of thrombolysis, through implementation of standardized protocols to reduce complications during thrombolysis treatment [11]. To this effect, some authors report systemic thrombolytic therapy to be the superior procedure in a standardized setting for selected patients [12]. This is in line with a 2016 study of Oezpeker et al. of 50 patients, favoring systemic thrombolysis therapy over surgical LVAD exchange [13]. Thus, the optimal management strategy currently remains an individual case by case decision, depending on the distinct experience of the center, and the decision should be made in a heart team, involving all disciplines involved.

This scenario becomes even more complex, if recurrent LVAD thrombosis takes place and literature on promising treatment strategies for recurrent LVAD thrombosis is totally lacking. To date, no study has been conducted on characteristics and prevalence of recurrent LVAD thrombosis, and nothing is known about risk factors or predictors accounting recurrent LVAD thrombosis. We report our experience in HeartWare patients with recurrent HVAD thrombosis and our standardized treatment strategy for these patients, favoring systemic thrombolysis therapy using t-PA.

This retrospective study summarizes characteristics, workflow, and clinical results of systemic thrombolysis treatment in recurrent HVAD thrombosis.

## Methods

### Data collection and follow-up

**Fig. 1:**
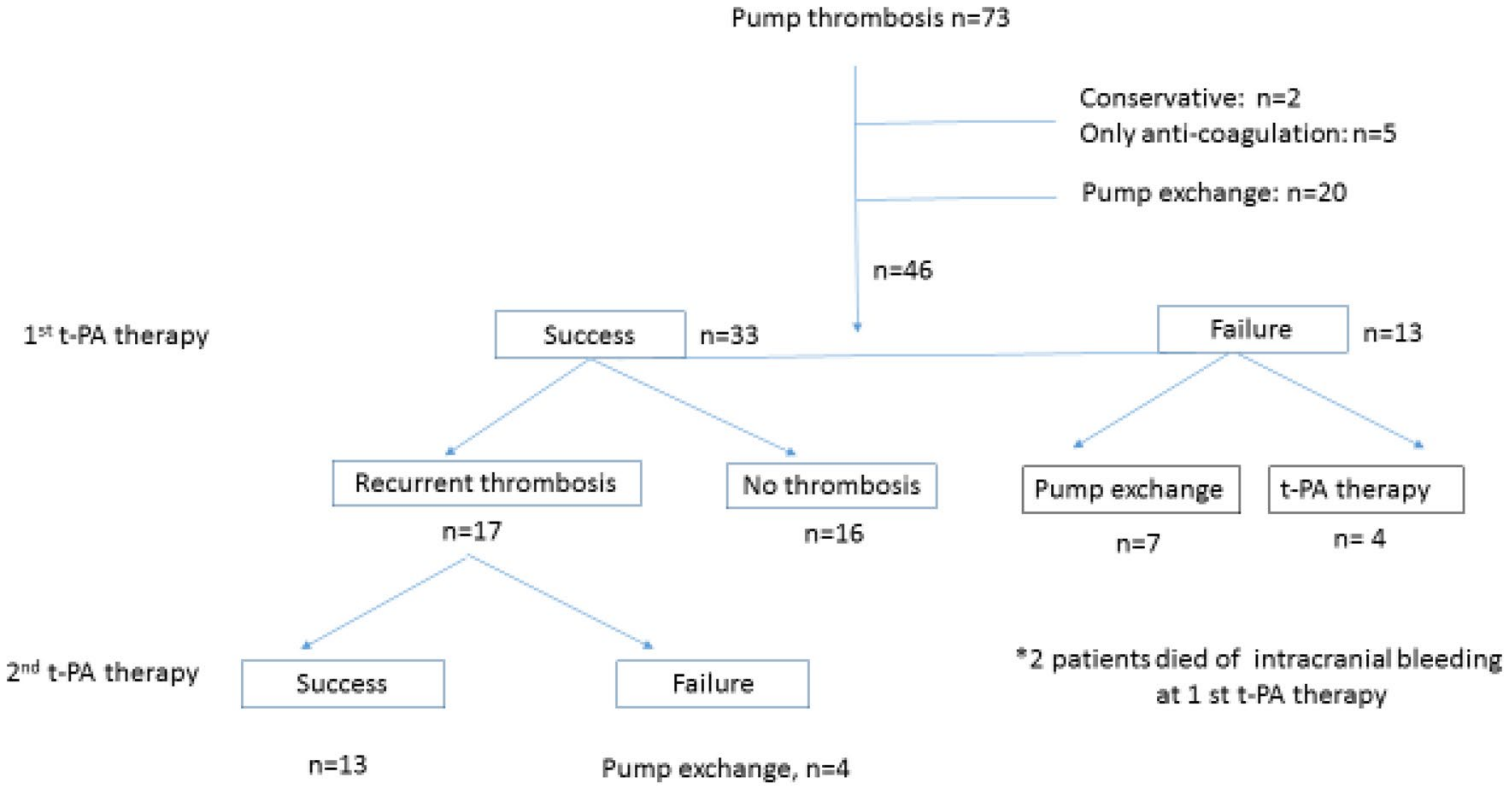
Patient selection enrolled in this retrospective study. *T*-*PA* tissue plasminogen activator

This single-center study was approved by institutional Ethics Committee of the Ruhr-University Bochum in Bad Oeynhausen (2020-619). We included all patients from our institutional database undergoing LVAD thrombolysis or surgical LVAD exchange between 2010 and 2019. Clinical decisions were made in our heart team conferences consisting of cardiologists, cardiac surgeons, perfusionists, cardio-anesthesiologists, psychologists, and VAD coordinators. Patients receiving biventricular assist devices or patients younger than 18 years of age were excluded from this analysis. Patients were included if they were treated with t-PA thrombolysis as initial treatment for LVAD thrombosis. If t-PA treatment was insufficient, a second thrombolysis attempt was considered or patients underwent surgical LVAD exchange, individually depending on the particular clinical setting. When recurrent thrombosis was diagnosed during follow-up, t-PA treatment was the therapy of choice again and if t-PA application failed in cases of recurrent LVAD thrombosis, repeating t-PA therapy was considerable or patients underwent surgical LVAD exchange, again depending on the individual patient’s condition. Efficacy of t-PA therapy was defined as no remaining LVAD thrombus proof, normalization of laboratory parameters, and normalized LVAD log-profiles. Thrombolysis success was defined as freedom from repeated thrombolysis, surgical LVAD exchange, and mortality 30 days after t-PA therapy. Study endpoint additionally includes all-cause mortality and heart transplantation. Patient characteristics of this retrospective study are depicted in Fig. 1.

### Diagnosis of LVAD thrombosis and t-PA treatment

**Fig. 2:**
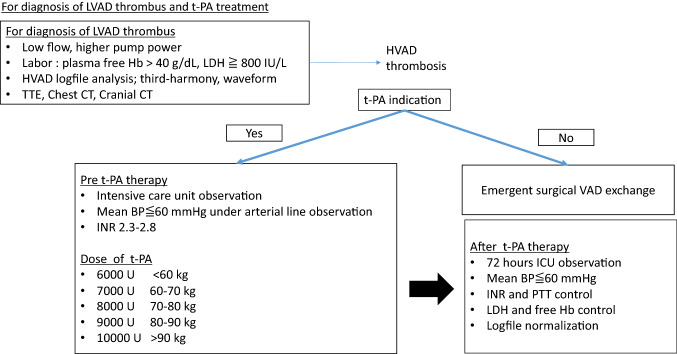
Our institutional standardized protocol for t-PA application. *LVAD* left-ventricular assist device, *t*-*PA* tissue plasminogen activator, *Hb* hemoglobin, *LDH* lactate dehydrogenase, *HVAD* HeartWare ventricular assist device, *TTE* transthoracic echocardiography, *CT* computed tomography, *BP* blood pressure, *INR* international normalized ratio, *PTT* partial thromboplastin time

LVAD thrombosis was suspected in the clinical setting of progressive symptoms of heart failure, LVAD low-flow alarm, laboratory parameters including hemoglobin, free-hemoglobin, lactate dehydrogenase, transthoracic echocardiography, contrast dye computed tomography, and failure of symptoms relief through intravenous volume application. Thresholds for LVAD thrombosis for lactate dehydrogenase are defined above 800 and quantitative plasma free-hemoglobin 15. Moreover, LVAD thrombosis was suspected through HVAD logfile readouts, when”third-harmony” occurred in the logfile, which is an established parameter suspecting impeller imbalance. With the diagnosis of HVAD thrombosis confirmed, t-PA treatment (intravenous bolus) was applied and vital signs were closely monitored on intensive-care unit. In patients with hemodynamic instability or outflow and inflow thrombosis, the heart team was consulted to consider surgical LVAD exchange individually (Fig. 2).

### Our institutional standardized protocol for t-PA application

We apply a standardized operating protocol when t-PA is administered (Fig. 2). In this protocol, uninterrupted patient surveillance and vital sign monitoring are implemented on intensive-care unit. Patients stay on intensive care regularly for 2 days when t-PA is applied. Before t-PA application, routinely cranial CT is performed in all patients to identify contraindications for t-PA use, such as intracranial bleeding or recent stroke. All patients are equipped with invasive hemodynamic monitoring and mean blood pressure is appointed to no more than 60 mmHg. All patients receive neurology workup and neurologic test which are annually repeated to identify or follow any curtailments. Patients were discharged from intensive care only after clinical relief from augmented heart failure symptoms and normalization of laboratory parameters including hemoglobin, free-hemoglobin, lactate dehydrogenase, as well as normalization of LVAD logfile readouts.

### Statistical analysis

Results are expressed as mean ± standard deviation (SD) or as median +25th–75th percentile interquartile range for continuous variables, and frequency and percentage for categorical variables. Univariable comparisons were performed with Student’s unpaired *t* test for continuous normally distributed data. The Mann–Whitney U test and Wilcoxon signed-rank sum test were used for comparisons of non-parametric continuous data and Fisher’s exact test for categorical data. Rates of freedom from all-cause death and heart transplantation were generated using the Kaplan–Meier method, and comparisons were made using the stratified log-rank test. To identify independent variables of recurrent LVAD thrombosis, multivariable Cox proportional-hazard regression analysis was subsequently used, the results are expressed as hazard ratios (HRs) with 95% confidence intervals (95% CIs). Candidate covariates were chosen based on current medical knowledge. A *p* value of < 0.05 was considered statistically significant, and all reported p values are two-sided. All statistical analyses were performed using R (The R Project for Statistical Computing; The R Foundation).

## Results

Among our 534 patients implanted with continuous-flow LVAD (HeartWare HVAD, Medtronic, USA), LVAD thrombosis occurred in *n *= 73 (13.7%) after a median of 10 months (IQR; 6–21 months). Of those 73 patients, 46 patients (63%) received t-PA therapy and 33 patients reached our study definition of successful thrombolysis therapy. 13 patients had unsuccessful results (success rate; 71.7%), Seven patients underwent surgical LVAD exchange and four patients received an additional t-PA application after three days. Two patients received no further treatment for following intracranial hematoma (Fig. 1).

### Recurrent LVAD thrombosis

**Fig. 3:**
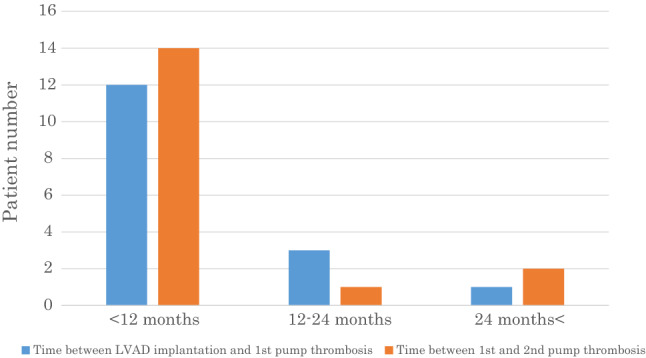
Time interval between 2 groups (LVAD implantation to first LVAD thrombosis and first to second LVAD thrombosis). *LVAD* left-ventricular assist device

Our 33 study patients with successful t-PA therapy were followed over a median of 13 months (IQR; 9–20 months) follow-up. During this follow-up, 16 patients remained free from further thrombotic events, but recurrent LVAD thrombosis occurred in 17 patients (51.5%) after 4.5 months (IQR; 3–6.8 months) follow-up. Patients with recurrent LVAD thrombosis were treated with t-PA therapy as the first-line therapy. 13 of those 17 recurrent LAD thrombosis patients had successful thrombolysis therapy as defined in this study, but 4 patients required surgical LVAD exchange due to unsuccessful thrombolysis. Heart transplantation was possible after a median of 14 months (IQR; 12–19 months). A flowchart of our study patients is provided in Fig. 1. We compared patient-baseline characteristics at LVAD implantation between patients that had developed recurrent LAD thrombosis vs. those without recurrent LAD thrombosis. No statistically significant baseline differences have been identified (Table 1), while only the time interval between initial LVAD implantation and first LVAD thrombosis is significantly shorter in patients with recurrent LVAD thrombosis, compared to those without recurrent LVAD thrombosis (*p* = 0.047) (Table 1). Within the first year after initial LVAD thrombosis, 14 patients (82.4%) developed a recurrent LVAD thrombosis (Fig. 3) and the time interval between LVAD thrombosis recurrence shortens with every thrombosis event (median 5 months, IQR; 3–9 months), compared to the time interval between LVAD implantation and first LVAD thrombosis (median 10 months, IQR; 8–15 months) (*p *= 0.046).

**Table 1:** Baseline characteristics

Variable	Recurrent pump thrombus (*n* = 17)	Non-recurrent pump thrombus (*n* = 16)	*p* value
Age at LVAD implantation	49 ± 13	51 ± 13	0.67
Male	15	13	0.66
Body mass index	26 ± 4.8	28 ± 8.2	0.41
Outpatient clinic systolic blood pressure	102 ± 15	100 ± 10	0.66
Outpatient clinic diastolic blood pressure	79 ± 13	78 ± 9.1	0.89
Pathology			0.53
Dilated cardiomyopathy	4	6	
Myocarditis	1	2	
Ischemic etiology	10	8	
Myocardial infarction	2	0	
INTERMACS			0.75
class1	6	5	
class2	6	8	
class3	5	3	
Hemodialysis dependent	0	1	0.49
Diabetes mellitus	6	3	0.44
Peripheral artery disease	1	0	1
Surgical history	1	3	0.34
Prior ECLS use	3	2	1
Temporary right ECLS use	3	3	1
International normalized ratio at first event	2.47 ± 0.42	2.31 ± 0.69	0.45
Thrombocytes at 1^st^ event, *10^4^ μl	18.4 ± 7.1	15.1 ± 7.0	0.24
Medication			
Acetylsalicylic acid	14	13	1
Heparin	4	2	0.66
Clopidogrel	2	0	0.49
Vitamin K antagonist	17	17	1
Period LVAD implantation and first pump thrombosis	Median 10 months (IQR; 8–15 months)	Median 19 months (IQR; 11–21 months)	0.047
Period first pump thrombosis and second pump thrombosis	Median 4.5 months (IQR; 3–6.75 months)		
Pump speed at discharge after HVAD implantation (rpm)	2688 ± 184	2700 ± 125	0.85
Pump power at discharge after HVAD implantation (Watt)	4.18 ± 0.91	4.36 ± 0.71	0.58
Pump speed at first pump thrombosis (rpm)	2734 ± 214	2750 ± 71	0.82
Pump power at first pump thrombosis (Watt)	5.75 ± 1.68	7.12 ± 5.29	0.26

*LVAD* left-ventricular assist device, *INTERMACS* Interagency Registry for Mechanical Assisted Circulatory Support, *ECLS* extracorporeal membrane oxygenation, *HVAD* HeartWare device

Multivariable Cox proportional-hazard regression analysis at a median of 29-month follow-up (IQR;16–52 months) identified the duration between LVAD implantation and emerging first LVAD thrombosis to be an independent risk factor for recurrent pump thrombosis (HR 0.93, 95% CI 0.87–0.99, *p *= 0.031) (Table 2). The first event was frequently caused within 12 months after LVAD implantation. The majority of secondary LVAD thrombosis occurred within 12 months after the first implantation.

**Table 2:** Cox proportional-hazard regression analysis for recurrent pump thrombosis

Predictors	HR	95% CI	*p* value	HR	95%CI	*p* value
Recurrent pump thrombosis	Univariate predictors			Multivariate predictors		
Age at implantation	0.99	0.95–1.04	0.76			
Body mass index	0.94	0.86–1.04	0.23			
INTERMACS	1.86	0.76–4.54	0.17			
Temporary right ECLS use	1.69	0.99–6.85	0.50			
Duration between LVAD imp and first pump thrombosis	0.91	0.85–0.98	0.018	0.93	0.87–0.99	0.031

*INTERMACS* Interagency Registry for Mechanical Assisted Circulatory Support, *ECLS* extracorporeal membrane oxygenation, *LVAD* left-ventricular assist device

### Survival

**Fig. 4:**
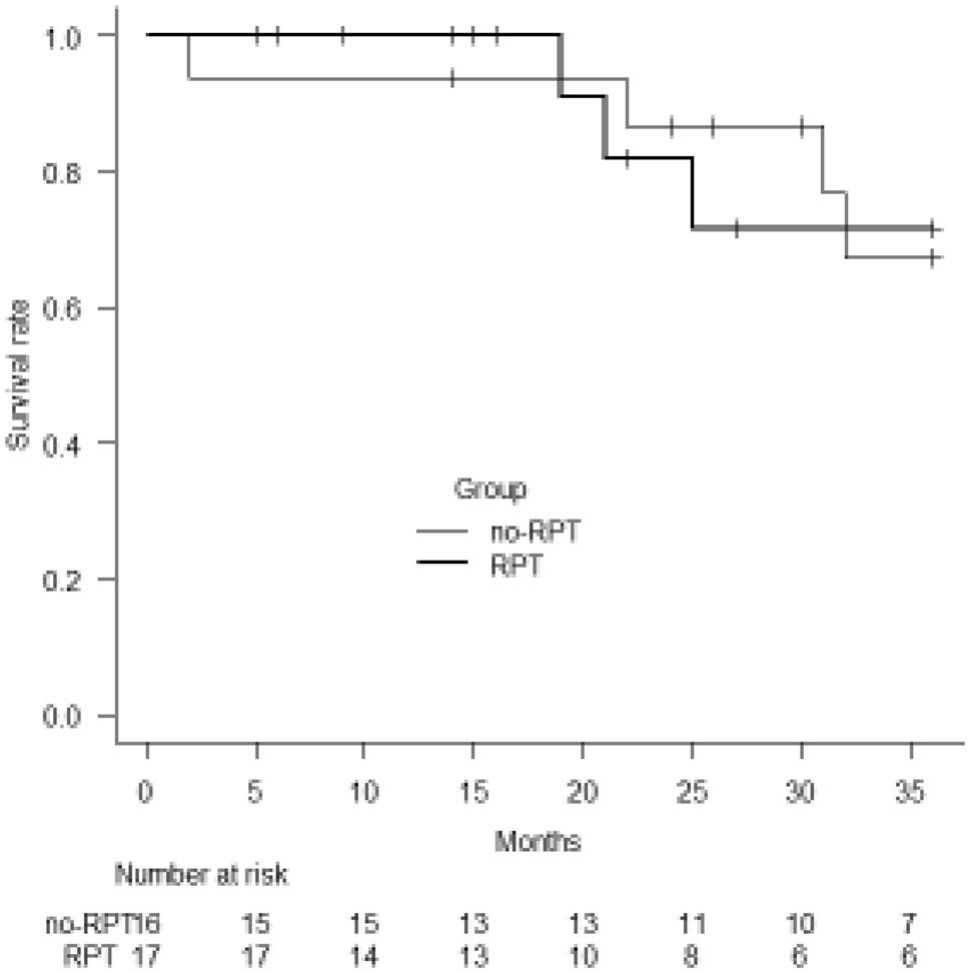
Kaplan–Meier curves for freedom from all-cause death for no recurrent LVAD thrombosis (thin line) vs. recurrent LVAD thrombosis (thick line). *RTB* repeat pump thrombosis

In this study freedom from all-cause death and heart transplantation was not significantly different over 3 years of follow-up after the first LVAD thrombosis event, for both patients with recurrent LVAD thrombosis versus those without (log-rank test: *p *= 0.96) (Fig. 4). In patients with recurrent LVAD thrombosis, two patients died from intracranial bleeding and infection at 11 months and 14 months after the second thrombolytic therapy. Moreover, six patients subsequently received heart transplantation without any complication. Survival rate at 1 year after t-PA therapy for recurrent LVAD thrombosis was 84.6% (95%CI 51.2–95.9%) according to Kaplan–Meier analysis (Heart transplantation was censored). Meanwhile, in the group of patients without recurrent LVAD thrombosis, one patient died of multiorgan failure two 2 months after t-PA therapy and heart transplantation was performed at 30 months in another patient in this group. Three patients died after 22 months for ICB, 31 months for cancer, and after 56 months for an unknown reason, respectively. Three other patients died from multiorgan failure after 32, 38, and 58 months.

### Cases of unsuccessful second t-PA therapy

Four patients (23.5%) had failure of t-PA therapy for their recurrent LVAD thrombosis and these patients underwent surgical LVAD exchange receiving a second HVAD (Medtronic, Fridley, MN, USA). All four patients subsequently underwent heart transplantation at 4, 9, 14, and 24 months, respectively, because of their contemplated very high risk of another recurrent LVAD thrombosis.

## Discussion

This is the first study to report characteristics, risk factors, predictors, and outcomes in patients with recurrent LVAD thrombosis in a large cohort. We demonstrate t-PA therapy to be a justifiable, safe therapy in recurrent LVAD thrombosis, with good success rates and low incidence of complication when conducted in a standardized setting. In our study, 73 patients (13.7%, n = 73/534) experienced LVAD thrombosis, and 46 patients (63%) received t-PA therapy at first LVAD thrombosis. Of the patients with a successful result, 17 patients (51.5%, n = 17/33) had developed a second HVAD thrombosis, all treated with t-PA therapy again. Our study did not identify any baseline characteristic to predict LVAD thrombosis recurrence, while we can show that the time interval until first occurrence of LVAD thrombosis is an independent predictor of recurrent LVAD thrombosis in the future. In addition to that, we demonstrate surgical pump exchange to be a feasible and arguable therapy in individual cases. Recurrent LVAD thrombosis occurs mainly within 12 months after the first pump thrombosis and the time interval between any further LVAD thrombosis shortens with every event. Survival rates in patient with and without recurrent LVAD thrombosis are comparable.

Various authors proclaim surgical LVAD exchange to be the gold standard of therapy in LVAD thrombosis [7, 14–16]. However, redo surgery per se is associated with increased post-surgery risk and mortality, especially in cohorts such as compromised LVAD patients. To reduce the risk of complications, thrombolysis therapy has recently been increasingly utilized in several centers, showing good results. When used in a standardized setting, systemic thrombolysis treatment with t-PA therapy can be first choice in LVAD thrombosis, while the decision for surgical LVAD exchange has to be made individually, preferably in patients with hemodynamic instability. T-PA therapy has serious risks including major bleeding, ischemic or hemorrhagic strokes, or incomplete thrombus solution that would require a second lysis attempt or surgical therapy. In addition, dosing and management algorithms related to t-PA administration in patients with LVAD thrombosis are uncertain, with no clear recommendations available. Therefore, with no universally accepted handling, t-PA administration depends on individual experience and the preferred strategy in the individual center [17, 18].

Nevertheless, LVAD thrombosis is a life-threatening condition and recommendations, and literature on promising therapy strategies is benevolently requested in daily clinical routine. Both t-PA administration and surgical treatment bear risks of bleeding and other complications and many clinical colleagues remain undetermined about the most promising therapy for their patients. This question becomes exceptionally eminent, in cases of recurrent LVAD thrombosis, because no trial has addressed this topic yet. Therefore, our study is meant to add to the current literature by reporting our experience of t-PA therapy in patients with recurrent LVAD thrombosis.

In our study, success rates of t-PA therapy between initial and recurrent thrombolysis are not statistically significant (71.7% vs.76.5%, *p *= 1.00). Interestingly, through t-PA thrombolysis for recurrent LVAD thrombosis, none of our study patients experienced serious complications such as bleeding, stroke, or death. We can conclude that t-PA therapy in recurrent LVAD thrombosis is as effective and safe as in cases of initial LVAD thrombosis and should be considered therapy of choice.

In a recent small retrospective series of 9 patients with initially successful therapy of LVAD thrombosis, 33% of the patients developed recurrent LVAD thrombosis. They found no associated mortality, while two patients experienced bleeding complications of non-fatal cerebral hemorrhage and minor bleedings [17]. In our study, success rate of t-PA therapy for the first and recurrent LVAD thrombosis was 71.7% and 76.5%, respectively, with fatalities of two patients died for intracranial hematoma after the first t-PA therapy.

Application of standardized procedures appears to increase patient safety and this study is designed to further improve our protocol (Fig. 2). All patients with pump thrombosis are treated on intensive-care unit and invasive blood pressure monitoring is essential to increase patient safety. Current recommendations declare to control mean blood pressure strictly below 60 mmHg. Through strict implementation of this strategy, we had no intracranial bleeding complication in our center during the recent years during t-PA treatment at all.

In a recent meta-analysis comparing thrombolysis therapy against surgical LVAD exchange, Luc et al. report surgical LVAD change to be superior in the aspect of survival and success of thrombus resolution. They even report lower rates of recurrent LVAD thrombosis [18]. On the other hand, a retrospective single-center study of 50 patients with HeartMate II and HVAD demonstrated similar survival at 2-year follow-up in patients receiving thrombolysis versus surgical LVAD exchange. However, their rates of thrombolysis failure and recurrent LVAD thrombosis were significantly higher in the thrombolysis group [13]. In our study, 33 HVAD patients had successful t-PA therapy at their first HVAD thrombosis and 17 patients required to repeat t-PA therapy for recurrent LVAD thrombosis. Moreover, our survival rate at 1-year follow-up is 100% (Fig. 3), while in our opinion, t-PA therapy could be associated with higher rates of recurrent LVAD thrombosis during follow-up, but repeated t-PA therapy is safe and sufficient, even in patients with recurrent LVAD thrombosis. In our study, four patients with recurrent LVAD thrombosis experienced insufficient thrombolysis therapy, requiring surgical LVAD exchange. All four patients survived surgery without any major complication in our study and all four patients received a new HAVD. All four patients had heart transplantation subsequently without major complication. According to recently published data by Schramm et al., the HeartMate 3 (Abbott, Abbott Park, IL, USA) has very low risk of LVAD thrombosis, which one could consider in case of recurrent LVAD thrombosis change the device into HeartMate 3 [19, 20].

To the best of our knowledge, no study has been conducted to investigate predictors of recurrent LVAD thrombosis yet and risk factors for recurrent LVAD thrombosis are completely unknown. Although enrolled the largest number of LVAD patients with recurrent LVAD thrombosis in the world, we have not been able to identify any patient characteristic or predictor in the context of recurrent LVAD thrombosis. Our analysis reveals only the time interval between initial LVAD implantation and incident of first LVAD thrombosis to be an independent risk factor for recurrent LVAD thrombosis in the future. We cannot explain at present why the first LVAD thrombosis occurs earlier in those patients with a recurrent thrombosis as the patient characteristics do not significantly differ between the groups. A conclusive assumption on this issue is limited because of the relatively small number of patients in this study and requires broader experience. We can only speculate whether this is based on non-adherence or yet unknown differences in patients’ coagulation system. Prevention measures of a secondary pump thrombosis include more frequent and adjusted control of anticoagulation therapy, however, with due consideration for the risk of bleeding. Weighing up the risk of bleeding against the risk of thrombus formation recurrence is a major challenge in the field of LVAD therapy and this topic has not been sufficiently studied so far. It is clear that patients with recurrent HVAD thrombosis are at higher risk of gastric bleeding or intracranial bleeding and complications in general. Therefore, additional studies are needed to address this complex and endangered patient population of HVAD recurrent thrombosis in the future.

Still, there are several limitations of our study. This is a retrospective, single-center study with a limited number of patients, although the largest in the world so far. Risk factors of LVAD thrombosis are multi-attribute, and for the information available in our study, we may have missed important information on various factors. Even though our study does not allow us to conclude generalizable recommendations, we were still able to report good outcomes in this complex patient collective on how to handle recurrent LVAD thrombosis. Future prospective multicenter studies with larger patient numbers are needed to further investigate this neglected topic.

We conclude that repeat t-PA therapy for recurrent pump thrombosis is safe and effective. In particular, in LVAD patients with high surgical mortality, repeat t-PA therapy may be a justifiable approach also for recurrent LVAD thrombosis.
